# Combination of Wnt/β-Catenin Targets S100A4 and DKK1 Improves Prognosis of Human Colorectal Cancer

**DOI:** 10.3390/cancers14010037

**Published:** 2021-12-22

**Authors:** Mathias Dahlmann, Anne Monks, Erik D. Harris, Dennis Kobelt, Marc Osterland, Fadi Khaireddine, Pia Herrmann, Wolfgang Kemmner, Susen Burock, Wolfgang Walther, Robert H. Shoemaker, Ulrike Stein

**Affiliations:** 1 Experimental and Clinical Research Center, a Cooperation between the Charité—Universitätsmedizin Berlin and the Max-Delbrück-Center for Molecular Medicine in the Helmholtz Association, Lindenberger Weg 80, 13125 Berlin, Germany; mathias.dahlmann@mdc-berlin.de (M.D.); dennis.kobelt@charite.de (D.K.); marc.osterland@fu-berlin.de (M.O.); fadykhaireddine@hotmail.com (F.K.); pia.herrmann@charite.de (P.H.); wolfgang.kemmner@charite.de (W.K.); wowalt@mdc-berlin.de (W.W.); 2 Molecular Pharmacology Laboratory, Leidos Biomedical Research, Inc., FNLCR, Frederick, MD 21702, USA; annemonks25@gmail.com (A.M.); erik.harris@nih.gov (E.D.H.); 3 Charité Comprehensive Cancer Center, Charité—Universitätsmedizin Berlin, Corporate Member of Freie Universität Berlin and Humboldt—Universität zu Berlin, Invalidenstraße 80, 10117 Berlin, Germany; susen.burock@charite.de; 4 Screening Technologies Branch, Developmental Therapeutics Program, Division of Cancer Treatment and Diagnosis, National Cancer Institute-Frederick, Building 440, Frederick, MD 21702, USA; shoemakr@mail.nih.gov; 5 German Cancer Consortium, 69121 Heidelberg, Germany

**Keywords:** S100A4, DKK1, Wnt signaling, colorectal cancer, patient survival

## Abstract

**Simple Summary:**

Aberrant Wnt/β-catenin signaling contributes to the development, progression, and metastasis of CRC, by altering target gene expression connected to cancer cell proliferation and motility. S100A4 is a Wnt/β-catenin target gene, which strongly enhances migration and invasion of CRC cells and thus CRC metastasis. Here, we report the transcriptional cross-regulation of S100A4 and the Wnt antagonist DKK1, in which the expression of S100A4 down-regulates DKK1 expression, sustaining activated Wnt signaling. S100A4 is an established prognostic biomarker for CRC patient survival, and the combination of S100A4 and DKK1 can be used to improve the prognosis of overall and metastasis-free survival.

**Abstract:**

Metastasis is directly linked to colorectal cancer (CRC) patient survival. Wnt signaling through β-catenin plays a key role. Metastasis-inducing S100A4 is a Wnt/β-catenin target gene and a prognostic biomarker for CRC and other cancer types. We aimed to identify S100A4-dependent expression alterations to better understand CRC progression and metastasis for improved patient survival. S100A4-induced transcriptome arrays, confirmatory studies in isogenic CRC cell lines with defined β-catenin genotypes, and functional metastasis studies were performed. S100A4-regulated transcriptome examination revealed the transcriptional cross-regulation of metastasis-inducing S100A4 with Wnt pathway antagonist Dickkopf-1 (DKK1). S100A4 overexpression down-regulated DKK1, S100A4 knock-down increased DKK1. Recombinant DKK1 reduced S100A4 expression and S100A4-mediated cell migration. In xenografted mice, systemic S100A4-shRNA application increased intratumoral DKK1. The inverse correlation of S100A4 and DKK1 was confirmed in five independent publicly available CRC expression datasets. Combinatorial analysis of S100A4 and DKK1 in two additional independent CRC patient cohorts improved prognosis of overall and metastasis-free survival. The newly discovered transcriptional cross-regulation of Wnt target S100A4 and Wnt antagonist DKK1 is predominated by an S100A4-induced Wnt signaling feedback loop, increasing cell motility and metastasis risk. S100A4 and DKK1 combination improves the identification of CRC patients at high risk.

## 1. Introduction

Colorectal cancer (CRC) is a major cause of cancer death worldwide and particularly in Western countries [1]. Turning healthy epithelial colon cells into CRC cells is frequently caused by increased Wnt signaling [2,3,4,5]. In sporadic CRC, somatic mutations in the *Adenomatous Polyposis Coli* (APC) are found in 70–80% of patients [6]. APC truncation distinctly reduces the degradation of β-catenin, which subsequently accumulates in the nucleus and triggers Wnt/β-catenin target gene expression even without upstream activation of the signaling pathway [7,8]. Similarly, aberrantly activated Wnt/β-catenin signaling is mediated by gain-of-function mutations in β-catenin itself, which occur in almost 50% of CRC tumors without APC mutations [9]. The majority of gain-of-function mutations (amino acid substitutions or in-frame deletions) within β-catenin occur in exon 3 at a regulatory region (aa32-aa45) for protein phosphorylation and binding of the E3 ubiquitin-protein ligase β-TrCP, which triggers the subsequent proteasomal degradation of β-catenin, resulting in a stabilization of mutated β-catenin in these cells [10].

One of the Wnt signaling target genes in CRC is the metastasis-inducing small Ca^2+^ binding protein S100A4 [11,12]. Its high abundance in tumor tissue, both intracellular and in the interstitial fluid, increases the metastatic potential of CRC cells and decreases overall survival (OS) and metastasis-free survival (MFS) of patients [13,14]. Therapeutic approaches to reduce S100A4 expression, and thereby restrict cancer progression and metastasis, including RNA interference (RNAi) [15,16,17] and small molecules for intervention strategies in the Wnt pathway [18,19,20,21].

Wnt/β-catenin signaling also regulates the expression of Dickkopf-1 (DKK1) [22]. DKK1 itself is an established Wnt antagonist, which competes to recruit Wnt co-receptors, such as LRP5/6, and is thus preventing the activation of Wnt signaling [23,24]. Elevated DKK1 expression in multiple myeloma and prostate cancer leads to enhanced bone metastasis [25,26,27], and the expression level of DKK1 affects the organotropism of breast cancer metastasis [28,29]. As an inhibitor of proliferative Wnt signaling, DKK1 has also been reported as a mediator to metastatic latency, where quiescent metastatic cells evade immune surveillance for later sporadic outgrowth [30]. Although DKK1 expression is associated with poor survival in many solid cancers, it is often found down-regulated in CRC and reports of its prognostic value in CRC metastasis are controversial [31,32,33].

In this study, we aimed to explore S100A4-induced transcriptome alterations in CRC cells. We discovered a so far undescribed feedback loop within the Wnt pathway. S100A4, the expression of which is induced by active Wnt/β-catenin signaling, suppresses the expression of DKK1. We analyzed this transcriptional cross-regulation of metastasis-inducing S100A4 and the Wnt antagonist DKK1 in CRC in cell culture and confirmed it in CRC xenografted mice. In addition, we found the activating transcription factor 5 (ATF5) involved in the expression regulation of DKK1 in CRC cells with restored low Wnt/β-catenin pathway activity. Combinatorial analysis of inverse S100A4 and DKK1 expression in human CRC patient specimens improved prognostication for patient survival.

## 2. Materials and Methods

### 2.1. Cell Lines, Culture Conditions, and Treatment

The CRC cell line HCT116 (heterozygous gain-of-function Δ45-β-catenin), and the single allele derivatives HAB68 (Δ45-β-catenin) and HAB92 (wild-type β-catenin), were kindly provided by Todd Waldman (Georgetown University, Washington, DC, USA). Plasmid transfection was performed with FugeneHD (Promega, Madison, WI, USA), according to the manufacturer’s instructions. Transfection of HAB92 and HCT116 cells with pcDNA3.1 or pcDNA3.1/S100A4 resulted in the control cell lines HAB92/vector and HCT116/vector, and in the S100A4 overexpressing cell lines HAB92/S100A4 and HCT116/S100A4, respectively. Transfection of HCT116 cells with S100A4-specific (shS100A4) or unspecific control shRNA-plasmids (shCtrl) resulted in HCT116/shS100A4 and HCT116/shCtrl cells, respectively. HAB92/shDKK1 and HAB92/shCtrl cells were generated by transfecting DKK1-specific (shDKK1) or unspecific (shCtrl) shRNA-plasmids (all SABiosciences, Frederick, MD, USA). Generated cell lines with modulated target gene expression were generated from clonal expansion of selected stably resistant cells after transfection.

Cell lines SW620, LS174T, Colo205, SW480, HCT15, LoVo, HT29, Caco 2, WiDr, KM12, SW48, and DLD-1 were obtained from ATCC. Authentication of the cell lines was verified by short tandem repeat (STR) genotyping at the DSMZ (German Collection of Microorganisms and Cell Cultures; Braunschweig, Germany). All cell lines were tested regularly for the absence of mycoplasma. The cell lines were cultured in recommended culture medium, supplemented with 10% FBS (all Life Technologies, Carlsbad, CA, USA). Cells were grown in sterile conditions in a humidified incubator (37 °C, 5% CO_2_, 95% humidity). Lyophilized recombinant human DKK1 (rDKK1) protein (R&D Systems, Minneapolis, MN, USA) was dissolved in PBS, supplemented with 1% BSA (Sigma, St. Louis, MO, USA). Cells were treated for 24 h with the indicated rDKK1 protein concentrations. Control cells were treated with the same amounts of 1% BSA solution alone.

### 2.2. Microarray Analysis of the S100A4-Induced Transcriptome

The competitive hybridization cDNA microarrays were performed at the Center for Cancer Research/NCI (National Cancer Institute, Frederick, MD, USA). Each experiment consisted of two identical microarrays containing reciprocally labeled cDNAs from test samples (HAB92/S100A4) and control samples (HAB92 or HAB92/vector, respectively), giving 4 arrays for analysis of expression differences. The test sample was stained with Cy5 (red fluorescence) and the control sample with Cy3 (green fluorescence). In reciprocal arrays, test samples were stained with Cy3 and controls were stained with Cy5. In brief, isolated total RNA from HAB92, HAB92/vector, and HAB92/S100A4 cells was reverse transcribed and labeled with either Cy5 or Cy3 dye. Test samples were combined with their respective control samples and hybridized onto Human OncoChip arrays (NCI). Fluorescence intensities were determined with a GenePix 4100A microarray scanner. The data were analyzed by GenePix Pro 4.1 software, and the microarray intensities were normalized by setting the ratio of medians to 1. Data were analyzed with available tools on mAdB (https://madb.nci.nih.gov) (accessed on 30 April 2011) and online tools from the ‘Database for Annotation, Visualization and Integrated Discovery’ (DAVID) [34,35].

### 2.3. Quantitative Real-Time RT-PCR (qRT-PCR)

Total RNA from cultured cells was extracted with Trizol RNA extraction reagent (Life Technologies). Total RNA from micro-dissected tumor tissues was isolated using the Universal RNA Purification Kit (Roboklon, Berlin, Germany). RNA was reverse transcribed as described previously [18]. cDNA quantification of target genes was performed with the following primers and probes: S100A4_fow: 5′-ctcagcgcttcttctttc-3′, S100A4_rev: 5′-gggtcagcagctccttta-3′, S100A4_FITC: 5′-tgtgatggtgtccaccttccacaagt-3′, S100A4_LCRed640: 5′-tcgggcaaagagggtgacaagt-3′; DKK1_fow: 5′-tagcaccttggatgggtattc-3′, DKK1_rev: 5′-agcctcctcctcacacctcctc-3′, DKK1_FITC: 5′-gtctccggtcatgagactgtgcc-3′, DKK1_LCRed640: 5′-aggattgtgttgtgctagacacctctgg-3′. The hG6PDH Kit (Roche, Basel, Switzerland) was used for cDNA quantification of the housekeeping gene G6PDH. Quantitative real-time RT PCR was performed in a LightCycler480 (Roche). Gene-specific standard curves and a calibrator in each run were used to quantify and normalize the samples. Each run was performed in duplicates and repeated at least twice with independent biological replicates.

### 2.4. Western Blot (WB) Analysis and Enzyme-Linked Immunosorbent Assay (ELISA)

Western blot analysis was performed as previously described [11]. Briefly, cells were lysed with RIPA buffer (Roche) for total protein extraction. Immunoblotting was performed with antibodies against hS100A4 (rabbit; 1:1000; Agilent, Santa Clara, CA, USA) and hGAPDH (goat; 1:1000; Santa Cruz Biotechnology, Dallas, TX, USA).

Secreted DKK1 was quantified with the DuoSet human DKK-1 ELISA System (R&D Systems). In brief, 4 × 10^5^ cells were plated into 6-well plates, and the cell-free supernatant was harvested after 24 h. The supernatant was diluted with blocking reagent (1:4 and 1:8; PBS with 1% BSA) before entering the previously blocked wells. rDKK1, dissolved in blocking reagent, was used as a standard. Each experiment was performed in duplicate with at least two different dilutions to assure that ELISA reaction occurred in the linear range of sensitivity. The mean values of secreted DKK1 from each supernatant were normalized to the amount of total protein extracted from the respective cells.

### 2.5. Chromatin Immunoprecipitation Assay (ChIP)

A total of 1 × 10^6^ cells were plated in 15 cm dishes 24 h prior to performing the assay. Cells were incubated with 13.5% formaldehyde for 10 min at room temperature to assure reversible cross-linking of proteins, washed twice with ice-cold PBS, and lysed according to the manufacturer’s instructions (Magna ChiP HiSens; Millipore, Burlington, MA, USA). Cell lysates were sonicated for 20 pulses at 100% output and centrifuged at 10,000 rpm at 4 °C for 10 min. The upernatant was transferred to a new tube and aliquoted in 50 μL aliquots for incubation with antibodies. A total of 5 μL of supernatant was stored at −20 °C and used as an input control. Each ChIP was incubated with 10 μg antibody or 10 μg control IgG overnight at 4 °C. Non-bound proteins were washed, followed by elution of the protein-DNA complex from the beads according to the manufacturer’s instructions. Cross-linking of protein and DNA was reversed, and PCR amplification of the DKK1-promoter was performed with a limit of 35 cycles, using the following primer set: pDKK1-fow: 5′-cgactaagcaagggagggg-3′; pDKK1-rev: 5′-gcctttataccgcgggcc-3′. PCR product was analyzed via 2% agarose gel electrophoresis.

### 2.6. Luciferase-Based Reporter Assay

For transient transfection, cells were seeded at a density of 4 × 10^4^ cells in a 24-well plate and directly transfected with 500 ng reporter plasmid using TransIT-2020 (MirusBio, Madison, WI, USA) according to the manufacturer’s instructions. After 48 h, cells were transfected with a mix of 100 ng DKK1-promoter plasmids (firefly luciferase) and 20 ng renilla luciferase plasmid (Promega) as an internal control. Cells were lysed 24 h after transfection, and reporter assay was performed using the Dual Luciferase Assay Kit (Promega) according to the manufacturer’s instructions. Firefly and Renilla luciferase activities were measured using an infiniteM200Pro (Tecan, Männedorf, Switzerland) plate reader.

### 2.7. Boyden Chamber Transwell Migration Assaya

Filter membranes of 12 μm pore size (Millipore) were used to analyze the migratory ability of HCT116/vector and HCT116/S100A4 cells. A total of 2.5 × 10^5^ cells were seeded into each transwell chamber, treated with 100 ng/mL rDKK1 or control solution, and incubated for 24 h to migrate through the membrane. After insert removal, cells at the bottom chamber were trypsinized and counted in a Neubauer chamber (LO Laboroptik, Friedrichsdorf, Germany). Each well was counted ten times. The experiments were performed in duplicates and repeated twice.

### 2.8. mRNA Expression Analysis of Xenograft CRC Mouse Tumor Tissue

Animal experiments were performed in accordance with the United Kingdom Co-ordinated Committee on Cancer Research (UKCCCR) guidelines and were approved by the responsible local authorities (State Office of Health and Social Affairs, Berlin, Germany), with the registration number A0010/19. mRNA samples of intrasplenically xenografted CRC mouse tumors were obtained after systemic treatment with shRNA expression plasmids. Experimental procedures were previously described [17]. In brief, NOD/SCID mice were intrasplenically transplanted with HCT116 cells and repeatedly treated with S100A4-shRNA expressing plasmids via tail vein injection. Mice were sacrificed, and spleens and livers were removed. Cryosections of the tumor tissue (spleen) were used to isolate total RNA samples.

### 2.9. Immunohistochemistry of Xenograft CRC Mouse Tumor Tissue

Cryosections of the tumor tissue (spleen) were fixed with 4% paraformaldehyde, quenched with 0.1 M glycine/PBS, and residual cellular peroxidase activity was blocked with 0.9% H_2_O_2_/PBS. Cells were permeabilized with 0.5% Triton-X-100/PBS and unspecific binding sites blocked with 1% horse serum/PBS. Target-specific antibodies (S100A4, Dako, 1:400; DKK1, CellSignalling, 1:400) were applied overnight in 0.1% horse serum/PBS and detected with a rabbit-specific DAB kit (Vectastain, Vector Laboratories, Inc., Burlingame, CA, USA) according to the manufacturer’s instructions. Nuclei were stained with hematoxylin, and pictures were taken with a BZ-X800 microscope (Keyence, Neu-Isenburg, Germany) at 20× and 40× magnification. Quantification of IHC signal intensities was performed with ImageJ (v1.53).

### 2.10. Data Mining of Expression Microarray Data

Publicly available expression data of CRC tumor microarrays were obtained from Gene Expression Omnibus (www.ncbi.nlm.nih.gov/geo) (accessed on 10 December 2018). Expression data of target genes were normalized to G6PDH and analyzed for direct or inverse correlation. Expression data of the following sets were combined after normalizing: GDS2201 [36]; GDS4381 [37]; GDS4513 [38]; GDS4515 [39]; and GDS4718 [40].

### 2.11. Patient Material

Primary tumors were obtained from all patients with informed written consent. The analyses of patient samples, in accordance with the Declaration of Helsinki, were performed with their consent to participate and were approved by the responsible local authorities (State Office of Health and Social Affairs, Berlin, Germany), with the registration number AA3/03/45. Primary tumor tissues were collected immediately after surgical removal and snap-frozen in liquid nitrogen according to internal protocols. In addition to routine pathological examination of the tumor tissue, the histopathology of each sample used for experimental analysis was reviewed by an experienced pathologist to confirm diagnosis, tissue composition, and tumor content. Tumor staging and typing were performed according to UICC and WHO guidelines. The patients were preoperatively untreated, had no history of familial CRC, did not suffer from a second tumor, and underwent R0 resection. One cohort consists of tumor samples from 41 CRC patients at stages I–IV. For the second cohort, CRC tumor tissues were obtained from 60 CRC patients at stages I, II, or III, i.e., without distant metastases at the time point of diagnosis. Detailed information on patients and tumor tissue of both cohorts are provided in previous reports [41,42]. All tumors were R0 resected, were fresh frozen in liquid nitrogen, and the areas of tumor cells were micro-dissected after preparation of serial consecutive cryosections.

### 2.12. Statistical Analysis

Student’s *t*-test was used to compare two groups of data. Comparison of more than two groups was performed by one-way analysis of variance (ANOVA) and Bonferroni post hoc multiple comparisons, or one-way ANOVA on ranks and Tukey post hoc multiple comparison, if the normality test of the data failed. Pearson correlation analysis was used to identify expression correlations. Survival rates were calculated with Kaplan–Meier estimator, with multiple comparisons (pairwise over strata) if indicated. The cut-offs to distinguish low and high expression levels were determined using Receiver–operator-characteristics (ROC) analysis by taking the value with the highest Youden-Index. *p*-values less than 0.05 were defined as statistically significant. All computations were performed using IBM SPSS Statistics 21.

## 3. Results

### 3.1. Inverse Expression Correlation of Wnt/β-Catenin Targets S100A4 and DKK1 in CRC Cells

#### 3.1.1. Identification of the S100A4-Induced Transcriptome

Although many protein–protein interaction partners of S100A4 have been identified that sustain the pro-metastatic action of S100A4 [5], much less is known about changes in the transcription of metastasis-associated genes upon elevated S100A4 expression level. To identify the transcriptional mechanism underlying S100A4-driven metastasis formation, we compared the expression profiles of the HCT116-derived isogenic cell lines HAB92, HAB92/vector, and HAB92/S100A4. HAB92 contains only the wild-type allele for β-catenin, resulting in reduced Wnt pathway activity and very low levels of S100A4 [11]. Ectopic overexpression of S100A4 in these cells was achieved by stable transfection of S100A4 cDNA. Competitive hybridization of cDNA from HAB92, HAB92/vector, and HAB92/S100A4 cells onto spotted microarrays identified 195 functionally annotated genes to be differentially expressed >4-fold on average (*n* ≥ 3).

**Figure 1:**
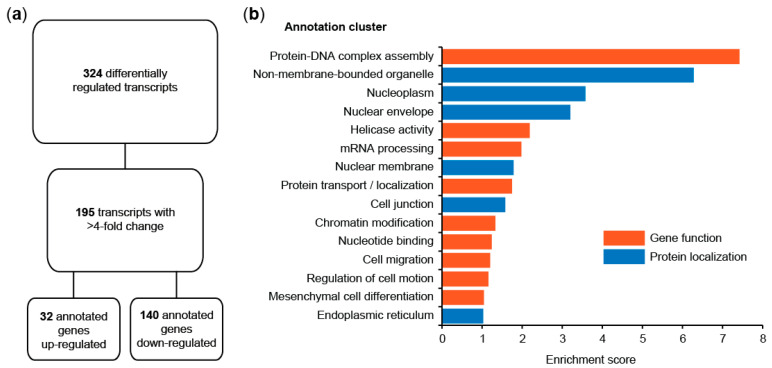
S100A4-dependent transcriptome analysis in HAB92 CRC cells. (**a**) Two color microarray analyses of differentially expressed genes in HAB92/S100A4 cells vs. HAB92 and HAB92/vector cells. (**b**) Annotation cluster analysis of 195 transcripts with a more than four-fold change in expression, classified by gene function and protein localization.

The results of four microarrays were combined and subsequently analyzed. A total of 324 transcripts were found differentially expressed in dependency of S100A4 overexpression, with 195 transcripts showing a more than four-fold difference. A total of 32 of the functionally annotated genes in this set were up-regulated, and 140 were down-regulated (Figure 1a). When we clustered the S100A4-associated genes according to their annotations using DAVID/EASE, we found high enrichment scores in topics related to transcription regulation (nuclear localization, DNA-protein complex assembly, chromatin modification, mRNA processing) and cell motility (Figure 1b). Interestingly, we observed a number of Wnt pathway factors and target genes differentially regulated in HAB92/S100A4 cells, indicating a previously unreported regulatory mechanism of S100A4 on Wnt signaling pathway activity (Appendix A).

#### 3.1.2. S100A4 Inhibits Expression of the Wnt Pathway Antagonist DKK1

**Figure 2:**
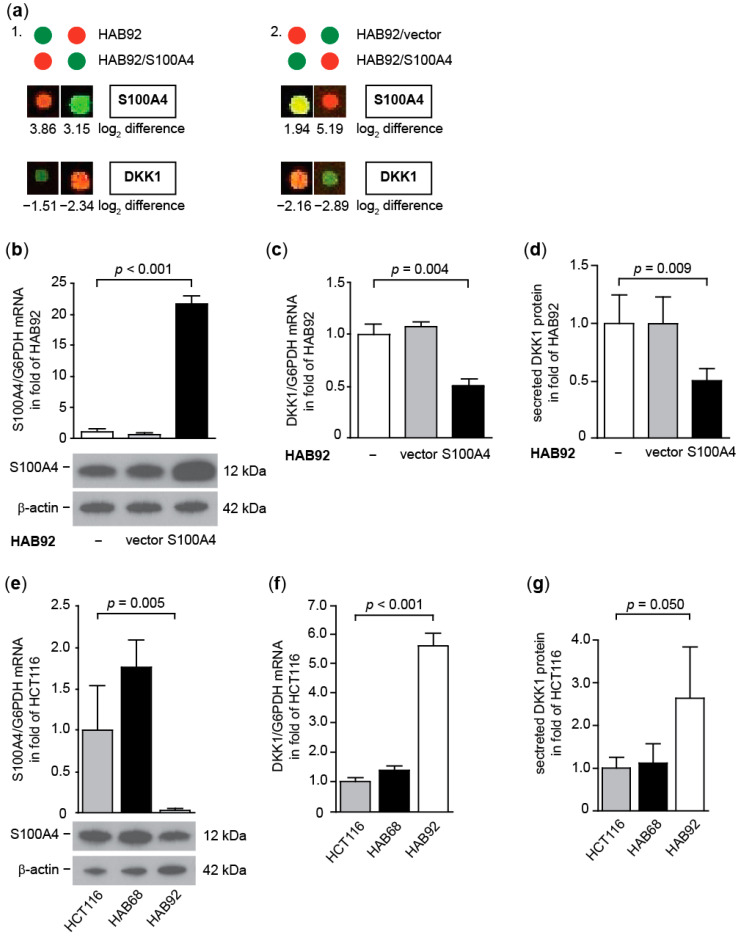
S100A4-induced expression alterations of DKK1 in CRC cell lines HCT116, HAB92, and HAB68. (**a**) Increased S100A4 expression and decreased DKK1 expression in HAB92/S100A4 cells vs. HAB92 cells (part 1) and HAB92/S100A4 cells vs. HAB92/vector cells (part 2). (**b**) Overexpression of S100A4 in HAB92/S100A4 cells on mRNA and protein level. Down-regulation of DKK1 mRNA expression (**c**) and of extracellular DKK1 (**d**) in HAB92/S100A4 cells. (**e**) S100A4 expression in HCT116, HAB68, and HAB92 cells on mRNA and protein levels; lowest S100A4 expression in HAB92 cells. Differential expression of DKK1 in HCT116, HAB68, and HAB92 cells on mRNA (**f**) and extracellular protein (**g**) levels; highest expression in HAB92 cells.

One of the most highly and consistently down-regulated genes in the arrays was DKK1 (Figure 2a). This result was validated by qRT PCR and WB. HAB92/S100A4 cells express 14.4-fold more S100A4 than HAB92 cells, both shown for mRNA and protein levels (*p* = 0.003; Figure 2b; Figure S1a,b), whereas mRNA levels of DKK1 were reduced to 40% in these cells, compared to HAB92 cells (*p* < 0.001; Figure 2c). By quantifying the amount of secreted DKK1 protein via ELISA, we observed a similar decrease to 34% in HAB92/S100A4 cells compared to HAB92 cells (*p* < 0.001; Figure 2d). These data confirm an S100A4-mediated decrease in DKK1. Although both S100A4 and DKK1 are target genes of canonical Wnt signaling, their expression pattern inversely differs in CRC tumors [22]. Therefore, we compared the levels of both genes in HCT116 cells, as well as in its derivatives HAB68 and HAB92. S100A4 expression was found to be 1.2-fold higher in HAB68 cells, harboring only mutant β-catenin, compared to HCT116 cells. In contrast, in HAB92 cells, with only wild-type β-catenin, S100A4 mRNA expression was reduced to 8% (*p* < 0.001; Figure 2e; Figure S1c,d). These data were supported by changes in protein expression, thereby confirming our previous finding [11]. In contrast, we observed an inverse expression pattern of DKK1 in those cells. HAB92 cells expressed nine-fold more DKK1 on the mRNA level compared to HCT116 cells (*p* < 0.001; Figure 2f). We validated the mRNA expression levels of DKK1 with the amount of secreted DKK1 protein in the surrounding medium and found a 5.3-fold increase of extracellular DKK1 from HAB92 cells, compared to HCT116 cells (*p* < 0.001; Figure 2g).

#### 3.1.3. Inverse Expression Correlation of S100A4 and DKK1 in Further CRC Cell Lines

**Figure 3:**
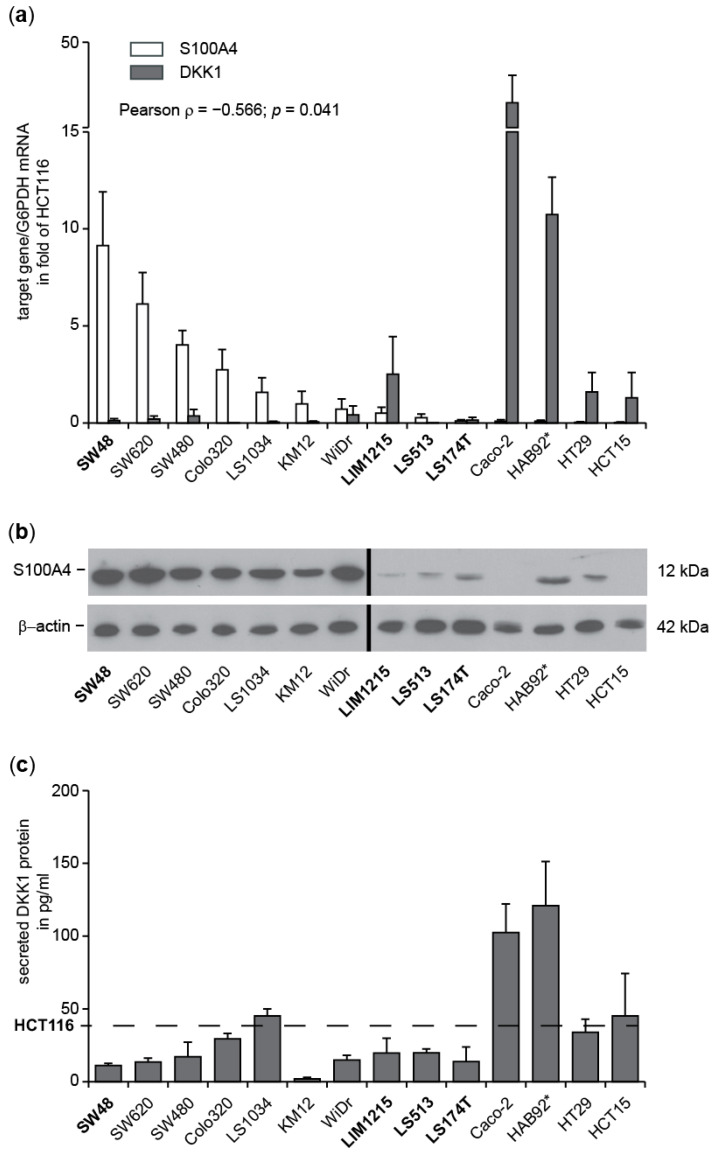
Inverse expression correlation of S100A4 and DKK1 in a panel of 13 CRC cell lines. (**a**) Relative mRNA expression level of S100A4 and DKK1 determined by gene-specific qRT PCR. (**b**) Western blot analysis of S100A4 expression. GAPDH served as loading control. (**c**) ELISA of extracellular amounts of human DKK1 in culture medium in the fold of HCT116. Names in bold indicate mutated β-catenin: HCT116—S45Δ, SW48—S33Y, LIM1215—T41A, LS513—A5-80Δ, LS174T—S45F. * indicates wt for both APC and β-catenin (Figure S1e–h).

In order to determine if the observation of inverse expression of S100A4 and DKK1 extended to other CRC cell lines, we compared the mRNA levels of both genes in a panel of 12 additional lines. A nearly reciprocal increase of either S100A4 or DKK1 expression was identified when normalized to HCT116 cells (Figure 3a). S100A4 mRNA levels in SW620, LS174T, Colo205, and SW480 cells were similar or higher than in HCT116 cells, and all these cell lines presented with very low levels of DKK1 mRNA. In contrast, HCT15, Lovo, HT29, HAB92, and Caco-2 cells expressed similar or higher levels of DKK1 than HCT116 cells and very low levels of S100A4 mRNA. The mRNA levels of both genes were low in WiDr, KM12, SW48, and DLD1 cells. The expression levels of S100A4 and DKK1 mRNA were significantly inversely correlated (Pearson correlation coefficient, ρ = −0.566; *p* = 0.041). The differences in mRNA expression levels were verified at the protein level by either WB for S100A4 (Figure 3b; Figure S1e–h) or ELISA for secreted DKK1 (Figure 3c).

#### 3.1.4. Expression Regulation of DKK1 in CRC Cells Involves the Transcription Factor ATF5

**Figure 4:**
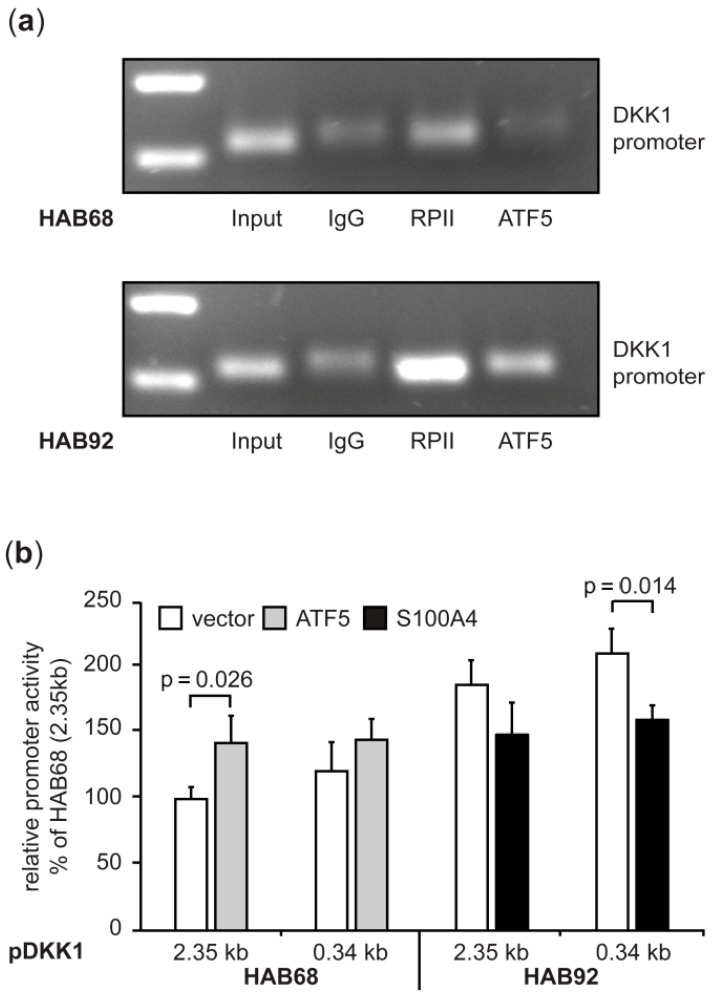
Expression regulation of DKK1 in CRC cells involves the transcription factor ATF5 and S100A4 but on different sites of the promoter. (**a**) ChIP assays of gene-specific pull-downs in HAB68 and HAB92 cells confirm the binding of ATF5 to the DKK1 promoter. Unspecific immunoglobulin served as the negative control and RNA polymerase II as an indicator of general DKK1 transcription. (**b**) Ectopic ATF5 expression in HAB68 cells increased DKK1 promoter-driven luciferase activity, while ectopic expression of S100A resulted in decreased reporter signal. DKK1 promoter fragments: −2238 bp–+112 bp (2.35 kb); −231 bp–+112 bp (0.34 kb).

By determining the S100A4-induced transcriptome in CRC cells, we were interested in which transcription factors are involved in the respective expression regulation of differentially expressed transcripts, focusing on the 140 down-regulated genes (Figure 1a). An in silico approach employed the iRegulon module in the Cytoscape analysis platform [43,44]. ATF5 was the only transcription factor predicted for DKK1 expression regulation (NES 3.37; *p* = 0.045), on the basis of ChIPseq data from Lovo cells (GSM1208713) [45]. We validated the binding of ATF5 to the DKK1 promoter via ATF5-specific ChIP assays in the HAB68 and HAB92 cell pair with differential expression of S100A4 and DKK1 (Figure 4a). We observed a higher abundance of DKK1 promoter fragments after precipitating RNA polymerase II in HAB92 cells, confirming the higher DKK1 transcription in these cells. In addition, precipitating ATF5 also resulted in increased band intensity after amplification of the DKK1-specific promoter fragment. To confirm the transcriptional regulation of DKK1 by ATF5 and S100A4 on promoter level, we used the previously described reporter plasmids containing truncated versions of the human DKK1 promoter [22]. We observed significantly increased luciferase reporter activity in HAB92 cells for all promoter fragments (Appendix B) and used the longest and shortest fragment for further analyses. Overexpression of ATF5 in HAB68 cells resulted in significantly increased reporter activity for the longest promoter fragment (2.35 kb; *p* = 0.026; Figure 4b), with a lesser extent for the shortest promoter fragment. In turn, ectopic expression of S100A4 in HAB92 did lead to a significant decrease in DKK1 promoter-driven luciferase activity (*p* = 0.014; Figure 4b).

#### 3.1.5. Transcriptional Cross-Regulation of DKK1 and S100A4 Affects S100A4 Phenotype

**Figure 5:**
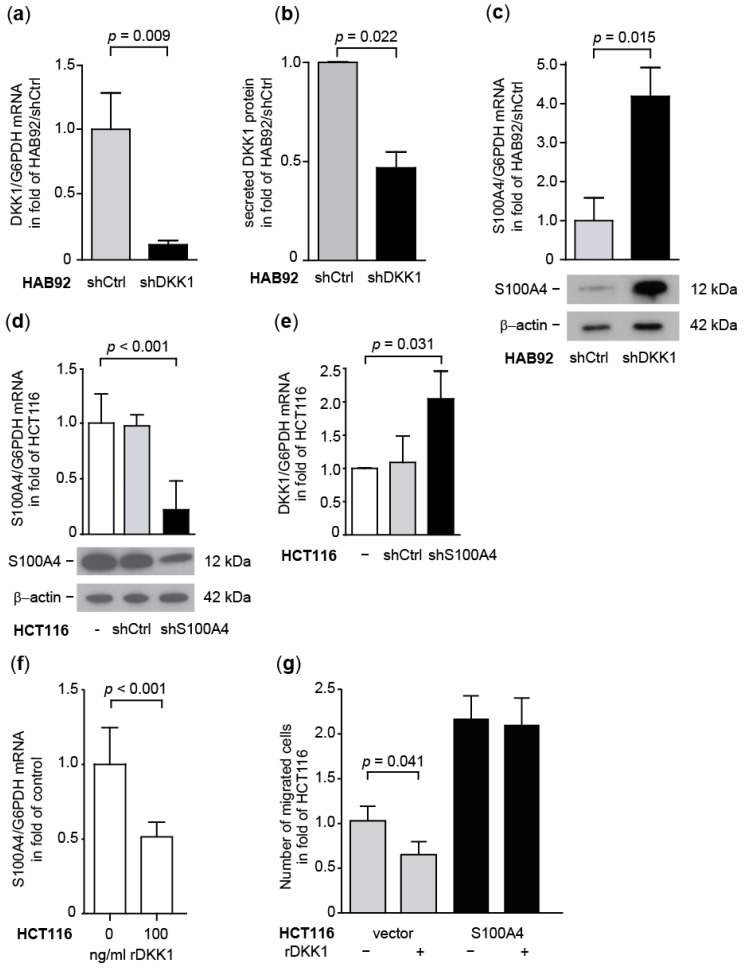
Transcriptional cross-regulation of DKK1 and S100A4 affects cellular motility. Relative DKK1 mRNA expression (**a**) and DKK1 protein secretion (**b**) in HAB92/shDKK1 cells. (**c**) Increase of S100A4 mRNA and protein expression in HAB92/shDKK1 cells. (**d**) Relative S100A4 mRNA and protein expression in HCT116/shS100A4 cells. (**e**) Increase of relative DKK1 mRNA expression in HCT116/shS100A4 cells. (**f**) Decrease of S100A4 mRNA expression level following treatment with rDKK1. (**g**) Decrease of cellular motility by rDKK1 treatment in HCT/vector cells is rescued by ectopic S100A4 expression in HCT116/S100A4 cells.

Since S100A4 overexpression inhibits the expression of DKK1 in CRC cells, we analyzed the functional consequences of this gene regulation. We transfected HAB92 cells with DKK1-specific shRNA plasmids, generating HAB92/shDKK1 cells. DKK1 mRNA expression in HAB92/shDKK1 cells was reduced to 9% of HAB92/shCtrl cells (*p* = 0.009; Figure 5a). The knock-down of DKK1 mRNA subsequently decreased the amount of secreted DKK1 protein in HAB92/shDKK1 cells to 47%, compared to the control cells (*p* = 0.022; Figure 5b). In turn, we observed a 4.2-fold increase in the S100A4 mRNA level of HAB92/shDKK1 cells, compared to control shRNA-transfected cells (*p* = 0.015) and an increase in S100A4 protein levels in HAB92/shDKK1 (Figure 5c; Figure S1i,j).

Next, we hypothesized that a reduction of S100A4 expression would result in increased expression of DKK1. We knocked down S100A4 in HCT116 cells by stably transfecting expression plasmids for either S100A4-specific shRNA (HCT116/shS100A4) or a non-targeting control shRNA (HCT116/shCtrl). HCT116/shS100A4 cells express 56% less S100A4 mRNA, compared to the control cells HCT116 and HCT116/shCtrl, which was confirmed at the protein level (*p* < 0.001; Figure 5d; Figure S1k,l). When we determined the expression of DKK1 in these cells, we observed a 2.0-fold increase in DKK1 mRNA levels in HCT116/shS100A4, compared to the control cells (*p* = 0.031; Figure 5e). Since DKK1 protein is secreted to exert its function as a Wnt pathway antagonist, we treated the HCT116 cells with rDKK1 and analyzed the S100A4 expression in those cells. Treatment with rDKK1 for 24 h reduced the S100A4 mRNA level in a concentration-dependent manner. Cells treated with 25 ng/mL rDKK1 expressed 85% (*p* < 0.01) less S100A4 mRNA, whereas treatment with 100 ng/mL rDKK1 reduced the S100A4 mRNA level to 68% (*p* < 0.001; Figure 5f). Treatment with rDKK1 also reduced cellular motility. Compared to untreated HCT116/vector cells, the migratory ability was diminished to 65% by the application of 100 ng/mL rDKK1 (*p* = 0.041; Figure 5g). Treatment with rDKK1 did not reduce the S100A4-mediated cell migration in cells with ectopic overexpression of S100A4.

#### 3.1.6. Knock-Down of Wnt Target Gene S100A4 Countermands Inhibition of DKK1

**Figure 6:**
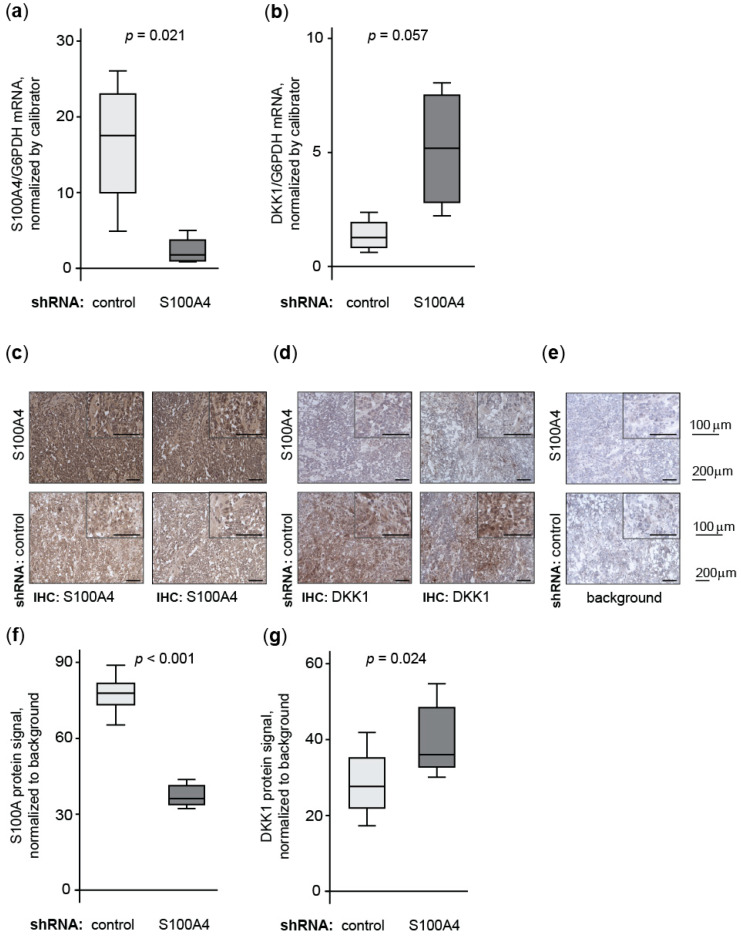
S100A4 reduction restores endogenous DKK1 expression in vivo. Relative mRNA expression of S100A4 (**a**) and DKK1 (**b**) in intrasplenic tumor tissue of xenografted mice receiving the systemic application of S100A4-specific shRNA expression plasmids. Immunostaining of S100A4 (**c**), DKK1 (**d**), and background control (**e**) of two independent samples per group of intrasplenic xenograft tumor tissue. Images were taken at 20× and 40× magnification, and scale bars represent 200 µm and 100 µm, respectively. Quantification of protein-specific immunostaining confirms the cross-regulation of S100A4 (**f**) and DKK1 (**g**) in vivo. Quantified expression of target genes occurred in triplicates of eight independent animal tumors.

To validate the transcriptional cross-regulation of S100A4 and DKK1 in vivo, we analyzed the S100A4-regulated DKK1 expression in tumor tissue of xenograft mice after intrasplenic transplantation of HCT116 cells. These animals were systemically treated with S100A4-specific shRNA expression plasmids (versus non-targeting control shRNA, [18]). We found significantly reduced S100A4 mRNA expression in tumor tissues of mice, treated with S100A4-specific shRNA plasmids (median = 1.78), compared to treatment with control shRNA plasmids (median = 17.52; *p* = 0.021; Figure 6a). Interestingly, we observed an inverse correlation for DKK1 mRNA expression levels when compared to S100A4 mRNA. Tumors of animals treated with S100A4-specific shRNA plasmids expressed increased levels of DKK1 mRNA (median = 5.19) compared to animals treated with control shRNA, showing only low DKK1 expression levels (median = 1.26; *p* = 0.057; Figure 6b). This result was confirmed by IHC, staining S100A4 and DKK1 protein in sequential cryo-sections of the tumor tissues (Figure 6c–e). After quantification of the protein signals, we determined a reduction in S100A4 protein in tumors treated with S100A4-specific shRNA plasmids (median = 36.69), compared to treatment with control-shRNA plasmids (median = 78.01, *p* < 0.001, Figure 6f). In turn, we found a significant increase of DKK1 protein expression in the tumor tissues with reduced S100A4 expression (median = 35.94), compared to control treatment (median = 27.54, *p* = 0.024, Figure 6g).

The reciprocal expression regulation of S100A4 and DKK1 in vivo was further validated by a lower abundance of human DKK1 in mouse plasma when ectopic S100A4 expression was induced in transplanted HAB92 cells (Appendix C).

### 3.2. Transcriptional Cross-Regulation of S100A4 and DKK1 Has Prognostic Value for CRC Patient Survival

#### 3.2.1. Inverse Expression Correlation of S100A4 and DKK1 in CRC Microarray Datasets

**Figure 7:**
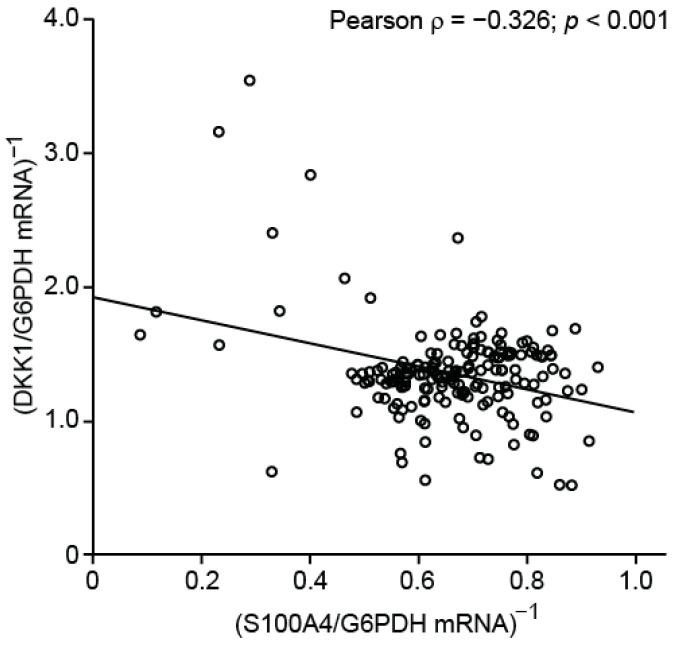
Correlation analysis of S100A and DKK1 mRNA expression of combined GEO datasets of CRC microarray analyses. Expression levels of target genes were normalized to G6PDH.

In order to evaluate the inverse expression correlation of S100A4 and DKK1 in patient tumors, we exploited several publicly available mRNA expression data generated by microarray analyses of CRC patient cohorts, using the GEO database from NCBI [36,37,38,39,40]. Expression values of S100A4 and DKK1 were normalized to G6PDH, and the five datasets were combined after normalization (*n* = 224). The inverse correlation of S100A4 and DKK1 mRNA expression in CRC patient tumors was confirmed by Pearson correlation analysis (ρ = 0.151; *p* = 0.024; Figure 7).

#### 3.2.2. Prognostic Value of Combining S100A4 and DKK1 Expression in CRC Tumor Samples

**Figure 8:**
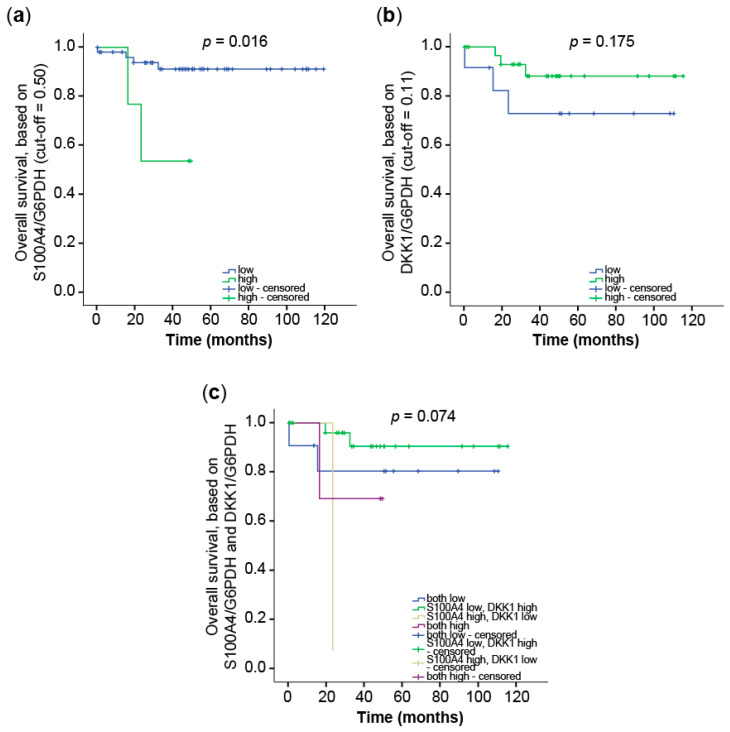
Combination of S100A4 and DKK1 for improved prognosis of OS CRC patients. DKK1, as well as S100A4, mRNA expression levels were determined by qRT PCR in micro-dissected tumor cell populations of primary tumors of stages I–IV (*n* = 41). The cut-off values to distinguish low and high expression levels were determined by ROC analyses. (**a**) OS of CRC patients, based on the S100A4 mRNA expression in the primary tumor. (**b**) OS of CRC patients, based on the DKK1 mRNA expression in the primary tumor. (**c**) OS of CRC patients, based on the combination of S100A4 and DKK1 expression in the tumor. Cut-off values for the respective gene and analysis are indicated by the axis labels.

Next, we determined the mRNA levels of S100A4 and DKK1 in micro-dissected primary tumor tissues of two independent patient cohorts by gene-specific qRT PCR. One cohort consisted of 41 CRC patients in stages I–IV [42]. Based on gene-expression levels and using ROC-based cut-off values, we calculated the rates for patients’ OS by Kaplan–Meier analysis. For S100A4, expression below the cut-off correlated significantly with better outcome in OS (*p* = 0.016; Figure 8a). The five-year OS was 90% (±4.6%) for low S100A4 expression and 50% (±2.5%) for S100A4 levels above the cut-off. On the contrary, patients benefited from higher expression levels of the Wnt antagonist DKK1 in longer OS (Figure 8b). The five-year OS was 87% (±7.0%) for high DKK1 levels and 71% (±14.3%) for low DKK1 expression.

**Figure 9:**
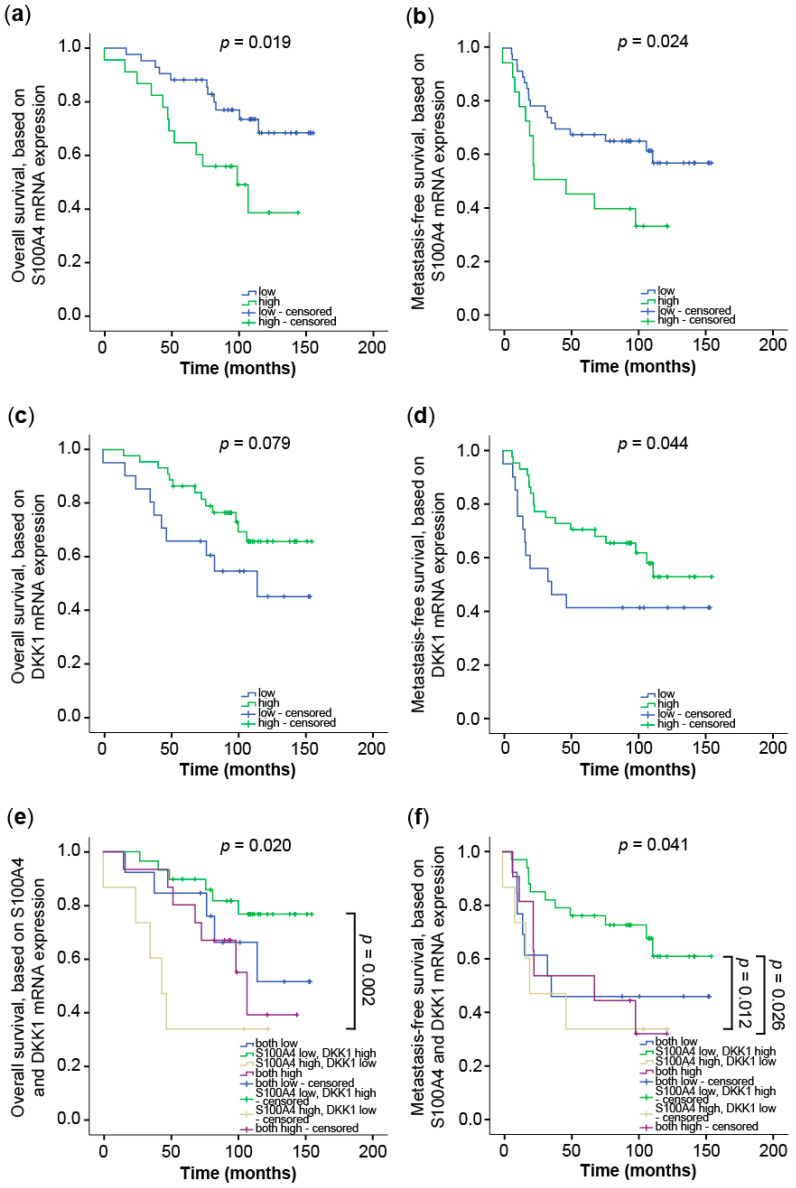
Combination of S100A4 and DKK1 for improved prognosis of OS and MFS of CRC patients. DKK1 and S100A4 mRNA expression levels were determined by qRT PCR in micro-dissected tumor cell populations of primary, not yet metastasized, tumors of stages I, II, and III (*n* = 60). The cut-off values to distinguish low and high expression levels were determined by ROC analyses (highest Youden index: S100A4—2.68; DKK1—0.21). Survival analysis was performed with the Kaplan–Meier estimator, with a chi-square multiple comparison. OS (**a**) and MFS (**b**) of CRC patients, based on the S100A4 mRNA expression in the primary tumor. OS (**c**) and MFS (**d**) of CRC patients, based on the DKK1 mRNA expression in the primary tumor. OS (**e**) and MFS (**f**) of CRC patients, based on the combination of S100A4 and DKK1 expression in the tumor.

The second cohort consisted of 60 CRC patients in stages I, II, and III (R0, no metastases at time of diagnosis) [41]. For S100A4, gene expression below the cut-off correlated significantly with better outcomes in both OS (Figure 9a) and MFS (Figure 9b). The five-year OS was 88% (±5.5%) for low S100A4 expression and 65% (±9.3%) for S100A4 expression levels above the cut-off. For MFS, the five-year survival rates were 65% (±7.3%) and 41% (±11.9%), respectively. On the contrary, patients benefited from higher expression levels of the Wnt antagonist DKK1 in OS (Figure 9c) and MFS (Figure 9d). The five-year OS was 63% (±11.1%) for low DKK1 expression and 85% (±5.5%) for high DKK1 levels. Likewise, the five-year MFS of 37% (±11.1%) for patients with low DKK1 levels increased to 68% (±7.3%) for DKK1 high expressers.

With the above-reported transcriptional cross-regulation of S100A4 and DKK1 expression in mind, we combined the survival analyses of S100A4 and DKK1 expression. The correlation for patients’ OS and their expression levels of the respective genes increased the significance when based on a combinatorial analysis of S100A4 and DKK1. If patients expressed low levels of S100A4 and high levels of DKK1 in the tumor, the five-year survival was 90% (±7.0%) in the first cohort (Figure 8c) and 91% (±6.1%) in the second cohort (Figure 9e). Expression of both genes below the respective cut-off resulted in a five-year survival rate of 79% (±13.4%) in the first cohort and 83% (±10.8%) in the second cohort. Patients with S100A4 and DKK1 expression above the respective cut-off resulted in a five-year survival rate of 67% (±27.2%) in the first cohort and 79% (±9.4%) in the second cohort. Patients with high S100A4 and low DKK1 expression levels showed the poorest five-year OS in both cohorts. No patient in the first cohort lived longer than five years, and the rate for the second cohort was 29% (±17.1%). When focused on MFS, the combination of S100A4 and DKK1 expression increased the significance with respect to DKK1 expression alone (Figure 9f). Patients expressing low levels of S100A4 and high levels of DKK1 showed a five-year MFS of 74% (±7.9%). Having both genes above the respective cut-off reduced the five-year MFS to 50% (±15.8%). If both genes are below the cut-off, a five-year MFS of 42% (±14.2%) was observed. High S100A4 and low DKK1 expression levels in the primary tumor resulted in a five-year MFS of 29% (±17.1%).

Taken together, the combination of S100A4 and DKK1 expression enables more powerful prognostication of patients’ outcomes. Patients with high S100A4 and low DKK1 expression levels in the primary tumor can be classified as high risk for OS. For MFS, patients with low intratumoral S100A4 expression become high-risk patients when the DKK1 expression is also decreased.

## 4. Discussion

Here we report the transcriptional cross-regulation of the Wnt target genes S100A4 and DKK1 by exploring the first S100A4-regulated transcriptome in CRC. Knock-down of S100A4 under constitutive active Wnt signaling restored the expression of DKK1 in vitro and in vivo. As overexpression of S100A4 reduced DKK1 mRNA and protein levels, S100A4 can be seen as the predominant factor in this feedback loop in Wnt signaling modulation. The inverse correlation of S100A4 and DKK1 was validated in publicly available CRC expression datasets. Combining the intratumoral expression levels of S100A4 and DKK1 increased OS and MFS prognostication and identification of CRC patients at high risk.

S100A4 expression is a marker for malignancy in several cancer types, including CRC [46,47]. Some effort has been made to understand the cellular mechanisms that regulate S100A4 expression. In CRC, the expression of S100A4 is mainly driven by constitutively active Wnt signaling [11]. By comparing transcripts of CRC cell lines that differed exclusively in the expression level of S100A4, we found that the Wnt antagonist DKK1 was inversely expressed. DKK1 itself is also a Wnt target gene, and it is up-regulated by highly active Wnt signaling [22,48]. Secreted DKK1 acts as a Wnt pathway antagonist by interacting with the membranous co-receptor LRP 5/6, which is subsequently sequestered from the Wnt/frizzled signaling complex [49,50]. This decrease in Wnt pathway activity creates a negative feedback loop in normal tissue. With our finding of high S100A4 levels upon active Wnt signaling, suppression of the pathway antagonist DKK1 should reduce the negative feedback loop allowing for sustained Wnt signaling. Indeed, the transcriptional up-regulation of DKK1 by active Wnt signaling has been lost in many cases of CRC [51,52,53]. The inverse correlation of S100A4 and DKK1 expression in CRC tumors was found significant when we analyzed the combination of several existing microarray datasets [36,37,38,39,40]. When we overexpressed S100A4 in CRC cells with wild-type β-catenin (HAB92), we observed a significant decrease in DKK1 expression along with AMOTL2, also described as a Wnt signaling inhibitor. Interestingly, we found a subset of other Wnt signaling target genes, such as CCND1, PTK2, and MET, down-regulated upon ectopic S100A4 expression in HAB92 cells with restored Wnt signaling pathway. A potential mechanism is the induced expression of APC itself, which can affect cytoplasmic and nuclear β-catenin levels, and thus, activity. In turn, a knock-down of DKK1 in these cells showed re-expression of endogenous S100A4. When we compared the transcriptional cross-regulation of S100A4 and DKK1 in cells harboring mutated β-catenin, the knock-down of S100A4 expression has a stronger effect on DKK1 expression than the treatment of the cells with 100 ng/mL rDKK1 on S100A4 expression. We conclude that S100A4 plays a dominant role in the regulation of DKK1 expression by preventing the normal negative feedback loop induced by DKK1, thus maintaining an activated Wnt pathway and stabilizing (or even increasing) its own expression level.

A recent publication by Park et al. describes remaining susceptibility to Wnt signaling pathway regulation by Wnt stimulation or APC regulation even in the presence of an S45Δ-β-catenin gain-of-function mutation, such as in HCT116 cells [54]. The proposed model of ‘just-right’ Wnt signaling activation in CRC cells is supported by our finding that S100A4 can modulate Wnt/β-catenin transcriptional activity even in the context of aberrantly active Wnt signaling.

We found ATF5, a member of the ATF/cAMP response element-binding protein family, involved in the regulation of DKK1 expression in CRC cell lines, depending on the activity of the Wnt/β-catenin signaling pathway. ATF5 itself has been related to cell enhanced invasion of fibrosarcoma and breast cancer cells [55], and its therapeutic targeting strongly reduced cancer cell survival, except for pancreatic cancer and CRC [56,57]. ATF5 is able to bind to CRE consensus sequences but prefers binding sites with a core sequence of CYTCTYCCTTW [58]. Interestingly, the promoter of GSK3β, a modulator of Wnt/β-catenin signaling activity, harbors a predicted ATF5 binding site, and it can regulate the levels of ATF5 itself [59,60]. With the here-reported regulation of DKK1 expression, ATF5 becomes further involved in the modulation of Wnt/β-catenin-mediated target gene expression, and thus cancer progression and metastasis also for CRC.

While constitutively active Wnt signaling in the colon gives rise to adenocarcinoma, elevated levels of S100A4 in the primary tumor drives cancer progression up to the formation of distant metastases [12,13]. S100A4 is, therefore, widely used as a prognostic marker to stratify patients’ risks to CRC [61,62]. In CRC, high DKK1 expression levels correlate with lower tumor stages, less metastasis, and increased five-year survival of patients [33,63,64].

The diagnosis of metastasized CRC is correlated with the worst prognosis for CRC patients [65]. With the reported transcriptional cross-regulation in expression regulation of S100A4 and DKK1, their combination in expression analyses should improve the prognostication for CRC patients. In our cohorts, patients with high S100A4 expression combined with low DKK1 expression showed the lowest five-year survival rates for both OS and MFS. High DKK1 expression in the tumor tissue or microenvironment seems to compensate for the aggressive phenotype of elevated S100A4 expression in OS. In the case of tumors with low S100A4 expression, patients’ outcome in MFS is strongly determined by the expression status of DKK1. The combination of both S100A4 and DKK1 clearly improves the prognostic value in CRC compared to each tumor marker alone.

Therapeutic approaches to restore the expression of DKK1 in tumors, with subsequent reduction of up-regulated Wnt target genes, combined with a reduction of S100A4 expression, could improve the outcome of S100A4-driven CRC. In recent reports, DKK1 expression in CRC cells was restored by treatment with Genistein or targeting the vitamin D receptor, leading to reduced Wnt target gene expression [66,67]. Further, pharmacological inhibitors, such as niclosamide and sulindac, are reported, which decrease S100A4 expression by intervening in the Wnt signaling, resulting in restricted metastasis formation [18,19].

## 5. Conclusions

Taken together, the identification of S100A4-mediated transcriptome revealed the transcriptional cross-regulation of the metastasis-inducing S100A4 and the Wnt antagonist DKK1, dominated by S100A4, which leads to increased cell motility, cancer progression and metastasis, and decreased survival of CRC patients. By combining both genes in expression analyses of CRC tumors, we were able to identify high-risk patients who might benefit from adapted cancer therapy.

## Figures and Tables

**Figure A1:**
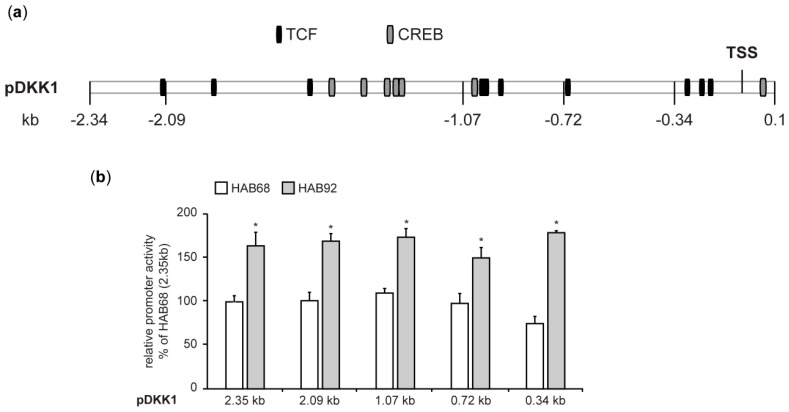
Expression regulation of DKK1 in HAB68 and HAB92 cells occurs near the transcription start site. (**a**) Graphical representation of the human DKK1 promoter. Predicted binding sites of transcription factor complexes containing TCF and CREB-family members are indicated in black and grey, respectively. (**b**) Relative DKK1 promoter-driven luciferase activity in HAB68 cells is significantly lower for all DKK1 promoter constructs compared to HAB92 cells. * statistically significant.

**Figure A2:**
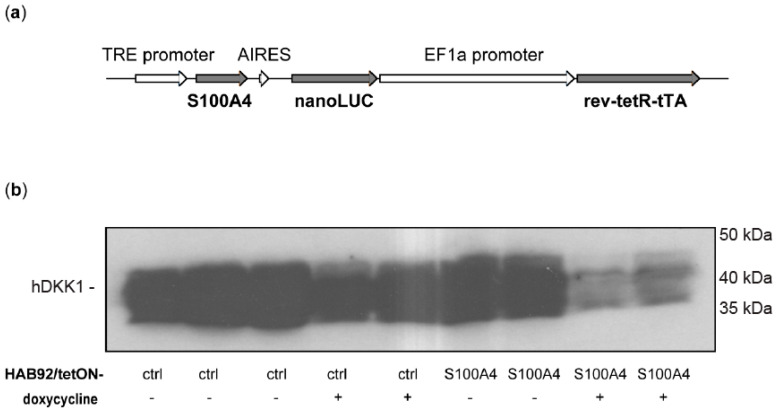
Induced ectopic expression of S100A4 in xenograft tumors results in a reduced abundance of human DKK1 in mouse plasma. (**a**) Schematic representation of the lentiviral vector for doxycycline-induced S100A4 expression. The respective control vector was generated without the coding sequence for S100A4 (not shown). Either vector was lentivirally transduced into HAB92 cells. (**b**) Plasma samples of mice intrasplenically transplanted with HAB92/tetON-S100A4 or HAB92/tetON-ctrl cells were taken after 15 days with or without doxycycline treatment. Immunostaining with human-specific DKK1 antibodies after WB showed reduced hDKK1 abundance in plasma samples of mice with induced S100A4 expression in the xenograft tumors.

## Data Availability

Data are available on request via the corresponding author.

## Supplementary Materials

The following are available online at https://www.mdpi.com/article/10.3390/cancers14010037/s1, Figure S1: Western Blots.

## Appendix A

**Table A1:** List of differentially expressed Wnt signaling pathway-related factors or target genes upon ectopic expression of S100A4 in HAB92 cells with restored Wnt signaling pathway activity.

log2-Fold Change	StDev	Gene Name	Description
5.04	1.21	APC	Promotes rapid degradation of β-catenin and participates in Wnt signaling as a negative regulator.
4.29	0.82	TBL1Y	Plays an essential role in transcription activation mediated by nuclear receptors.
−1.72	0.22	CAMK2D	Calcium/calmodulin-dependent protein kinase involved in the regulation of Ca^2+^ homeostasis.
−1.72	0.36	CD44	Cell-surface receptor that plays a role in cell–cell interactions, cell adhesion, and migration.
−1.73	0.36	HDAC2	Responsible for the deacetylation of lysine residues on the N-terminal part of the core histones (H2A, H2B, H3, and H4).
−1.84	0.57	CCND1	Regulatory component of the cyclin D1-CDK4 (DC) complex that regulates the cell cycle during G_1_/S transition.
−1.99	0.43	EPCAM	Plays a role in embryonic stem cells proliferation and differentiation.
−2.13	0.35	PTK2	Non-receptor protein-tyrosine kinase that plays an essential role in regulating cell migration, adhesion, formation, and disassembly of focal adhesions and cell protrusions, cell cycle progression, cell proliferation, and apoptosis.
−2.23	0.57	DKK1	Antagonizes canonical Wnt signaling by inhibiting LRP5/6 interaction with Wnt and by forming a ternary complex with the transmembrane protein KREMEN that promotes internalization of LRP5/6.
−2.26	0.22	MET	Receptor tyrosine kinase that transduces signals from the extracellular matrix into the cytoplasm by binding to hepatocyte growth factor/HGF ligand. Regulates many physiological processes, including proliferation, scattering, morphogenesis, and survival.
−2.50	0.80	AMOTL2	Regulates the translocation of phosphorylated SRC to peripheral cell-matrix adhesion sites. Inhibits the Wnt/β-catenin signaling pathway, probably by recruiting β-catenin to recycling endosomes, and hence preventing its translocation to the nucleus.
−5.22	3.81	TBL1XR1	F-box-like protein involved in the recruitment of the ubiquitin/19S proteasome complex to nuclear receptor-regulated transcription units. Plays an essential role in transcription activation mediated by nuclear receptors.

## Appendix B

To investigate in which region of the DKK1 promoter an ectopic expression of S100A4 and ATF5 in HAB92 and HAB68 cells, respectively, will affect DKK1 transcription, we employed previously reported luciferase reporter constructs, driven by DKK1 promoter fragments of different sizes (kind gift of Alberto Munoz; Gonzalez-Sancho et al., Oncogene 2005). A schematic view of the promoter fragments and their predicted binding sites of TCF and CREB-transcription factor family members is depicted in Figure A1a. Relative luciferase reporter activity from each DKK1 promoter fragment is displayed in Figure A1b. Luciferase activity is not significantly altered within each CRC cell line, but we observed significantly higher reporter activity in HAB92 cells compared to HAB68 cells (Figure A1b). As this reflects the observed difference in DKK1 expression between these two cell lines, we focused on the region around the transcription start site (0.34 kb fragment) in comparison to the longest fragment (2.34 kb).

## Appendix C

To confirm the expression regulation of DKK1 by ectopic expression of S100A4 in vivo, we generated a doxycycline-induced S100A4 expression vector (tetON-S100A4-AIRES-nLUC; Figure A2a) for lentiviral transduction. This vector and a control vector without the S100A4 coding sequence (tetON-ctrl-AIRES-nLUC) were transduced via lentiviral particles into HAB92 cells, generating HAB92/tetON-S100A4 and HAB92/tetON-ctrl cells, respectively. A total of 1 × 10^6^ cells of each cell line were transplanted into the spleens of NOG mice, and for half of each group, 6 mg/kg of doxycycline was supplied in the drinking water. Plasma samples were taken after 15 days, and equal protein amounts were analyzed for the abundance of human DKK1 via WB. We observed a distinct reduction of hDKK1 in the plasma samples of doxycycline-treated mice harboring HAB92/tetON-S100A4 cells, compared to untreated mice from the same group (Figure A2b; Figure S1m). Mice with transplanted HAB92/tetON-ctrl cells showed only a minor decrease of hDKK1 in their plasma after doxycycline treatment, which points to a mild unspecific treatment effect. The animal experiment was performed in accordance with the United Kingdom Co-ordinated Committee on Cancer Research (UKCCCR) guidelines and was approved by the responsible local authorities (State Office of Health and Social Affairs, Berlin, Germany), with the registration number G0030/15.
