# Supplementary figures and images for: Combination of Wnt/β-Catenin Targets S100A4 and DKK1 Improves Prognosis of Human Colorectal Cancer
Source: Cancers (Basel). 2021 Dec 22;14(1):37. doi: 10.3390/cancers14010037 (PMC8750436; doi:10.3390/cancers14010037)

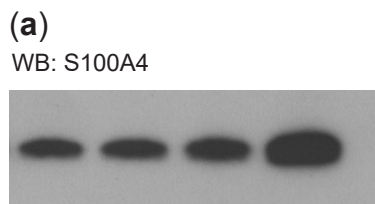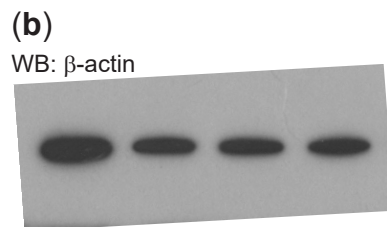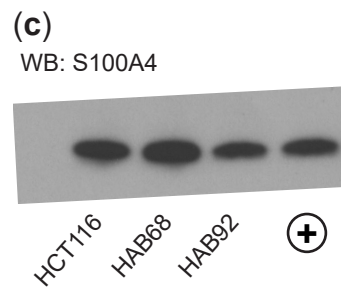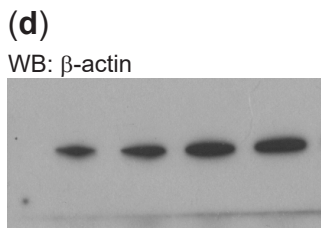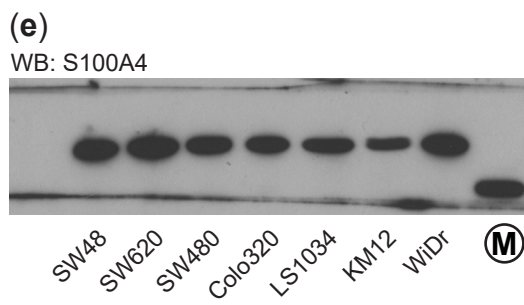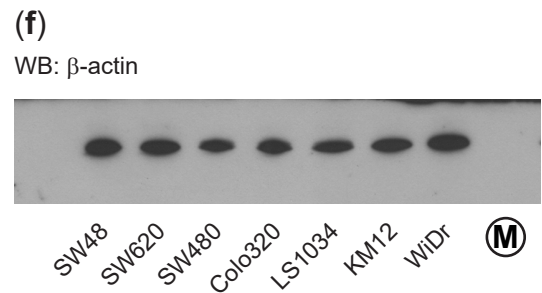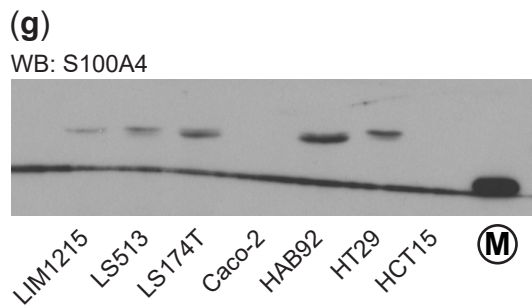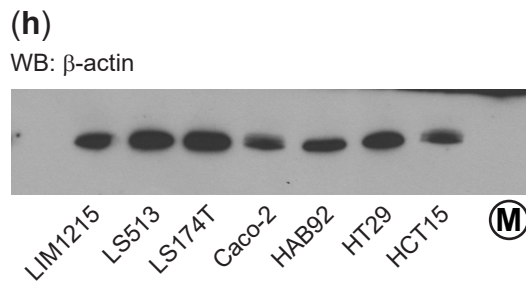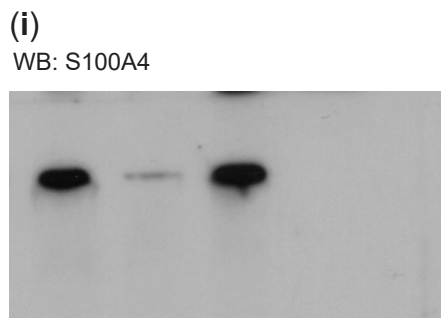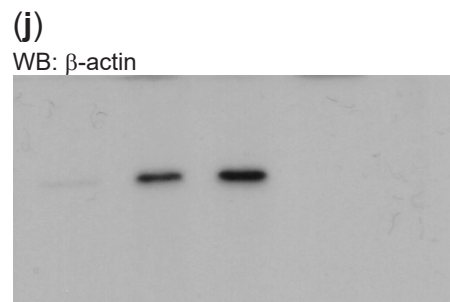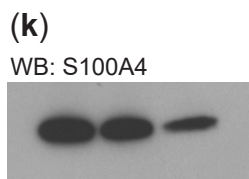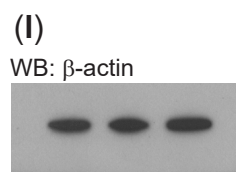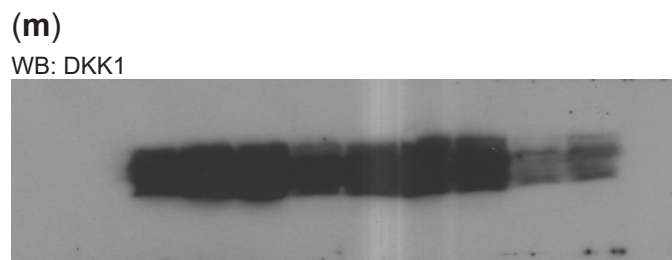

**(+)** – respective positive control

**(M)** – protein weight marker lane

Supplement: Supplementary file 1 [file cancers-14-00037-s001.zip › cancers-1432557-supplementary.pdf]
